# In Vitro Efficacy of Terpenes from Essential Oils against *Sarcoptes scabiei*

**DOI:** 10.3390/molecules28083361

**Published:** 2023-04-11

**Authors:** Meilin Li, Shenrui Feng, Siyi Huang, Jacques Guillot, Fang Fang

**Affiliations:** 1 Parasitology Department, College of Animal Science and Technology, Guangxi University, Nanning 530004, China; 2 Department of Pathogenic Biology, Army Medical University (Third Military Medical University), Chongqing 400038, China; 3 Dermatology-Parasitology-Mycology Departement, Oniris, 100 Route de Gachet, 44300 Nantes, France; 4 Guangxi Zhuang Autonomous Region Engineering Research Center of Veterinary Biologics, Nanning 530004, China; 5 Guangxi Key Laboratory of Animal Reproduction, Breeding and Disease Control, Nanning 530004, China

**Keywords:** *Sarcoptes scabiei*, scabies, terpenes, essential oils

## Abstract

The mite *Sarcoptes scabiei* is responsible for the emerging or re-emerging skin disease called scabies in humans and sarcoptic mange in animals. Essential oils represent an appealing alternative strategy for the control of *Sarcoptes* infections, but the commercial development of essential oils may be hampered by their inconsistency in efficacy due to their varied chemical compositions. In order to address this issue, we assessed the efficacy of six components (carvacrol, eugenol, geraniol, citral, terpinen-4-ol, and linalool) against *S. scabiei*. At a concentration of 0.5%, carvacrol presented the best miticidal efficacy, with a median lethal time (LT_50_) value of 6.7 min, followed by eugenol (56.3 min), geraniol (1.8 h), citral (6.1 h), terpinen-4-ol (22.3 h), and linalool (39.9 h). The LC_50_ values at 30 min for carvacrol, eugenol, and geraniol were 0.24, 0.79, and 0.91%, respectively. In conclusion, carvacrol, eugenol, and geraniol represent potential complementary or alternative agents for *S. scabiei* infections in humans or animals. Our study provides a scientific basis for the development of scabicidal products based on essential oils.

## 1. Introduction

The mite *Sarcoptes scabiei* is the causative agent of a highly contagious skin disease in humans and a large number of domestic and wild mammals [1,2]. The disease is characterized by irritation, inflammation, hyperkeratosis, alopecia, and pruritis, often accompanied by secondary infections [3,4]. Human scabies was added to the WHO list of neglected tropical diseases in 2017, with an estimated 200 million cases annually [5]. Additionally, *Sarcoptes* infections affect animal health and production, resulting in financial losses and animal welfare issues [6].

Controlling scabies is challenging as there are no vaccines available, and the two most commonly used broad-spectrum acaricides, ivermectin and permethrin, are becoming less effective due to increasing resistance [7]. Additionally, these acaricides do not kill eggs [8], which can lead to recurrent infections [9]. Therefore, the development of safer and more effective drugs against *S. scabiei* is required.

A large number of essential oils have been shown to be effective against *Sarcoptes scabiei* [10,11,12,13]. The essential oils of tea tree (*Melaleuca alternifolia*) [14], lemongrass (*Cymbopogon citratus*) [15], lavender (*Lavandula angustifolia* Mill.) [16], clove (*Eugenia caryophyllata*) [16,17], palmarosa (*Cymbopogon martinii*) [16], cinnamon (*Cinnamomum zeylanicum*), and tulsi (*Ocimum sanctum*) [18] are promising agents for scabies control. However, the results from in vitro studies using essential oils have not always been consistent due to their complex and varied compositions [19]. Therefore, single miticidal components should be tested so that they can be used at known concentrations and application rates. Previous studies have demonstrated that terpinen-4-ol, citral, linalool, eugenol, geraniol, and carvacrol possess good miticidal effects against *Psoroptes* mites [20,21,22]. Terpinen-4-ol and eugenol have been evaluated against *S. scabiei* [14,17], but the miticidal activities of citral, linalool, geraniol, and carvacrol have not been tested against *S. scabiei*.

In order to ensure the consistency and reliability of essential oil products and identify the most efficacious components for further research, we aimed to assess the in vitro miticidal activities of six essential oil components, namely terpinen-4-ol, citral, linalool, eugenol, geraniol, and carvacrol, against *S. scabiei*.

## 2. Results

**Figure 1:**
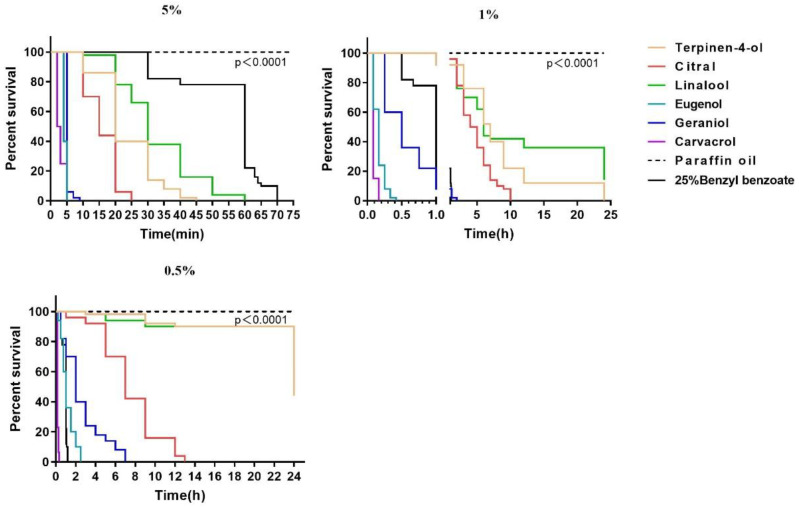
Survival curves of *Sarcoptes scabiei* mites exposed to 5%, 1%, and 0.5% solutions of six terpenes.

The six components, namely terpinen-4-ol, citral, linalool, eugenol, geraniol, and carvacrol, exhibited time- and concentration-dependent miticidal activity against *S. scabiei*. In all of the tests, significant statistical differences were found between each compound and the negative control (*p* < 0.0001). Based on the survival curves (Figure 1) in the contact bioassays, all of the terpenes killed all of the motile stages of the mites within 60 min at a concentration of 5% compared with the positive control of 25% benzyl benzoate, which killed 78.0% of the mites within 60 min. At a concentration of 1%, the average lethal time after exposure to carvacrol, eugenol, geraniol, citral, terpinen-4-ol, and linalool was 10 min, 17.6 min, 23.6 min, 8.4 h, 19.2 h, and >24 h, respectively. Carvacrol, eugenol, geraniol, and citral killed all of the mites within 24 h at a concentration of 0.5%, while the mite mortality rate was only 28% with linalool at the same concentration. Therefore, the efficacy of the six terpenes can be presented in the following order: carvacrol > eugenol > geraniol > citral > terpinen-4-ol > linalool. The LT_50_ values are presented in Table 1. After 30 min of immersion, carvacrol displayed the lowest LC_50_ value (0.26%), followed by eugenol (0.38%) and geraniol (0.56%) (Table 2). The negative control of paraffin oil displayed no miticidal activity against the motile stages of *S. scabiei* (Figure 1).

## 3. Materials and Methods

### 3.1. Sarcoptes scabiei Mites and Compounds

**Figure 2:**
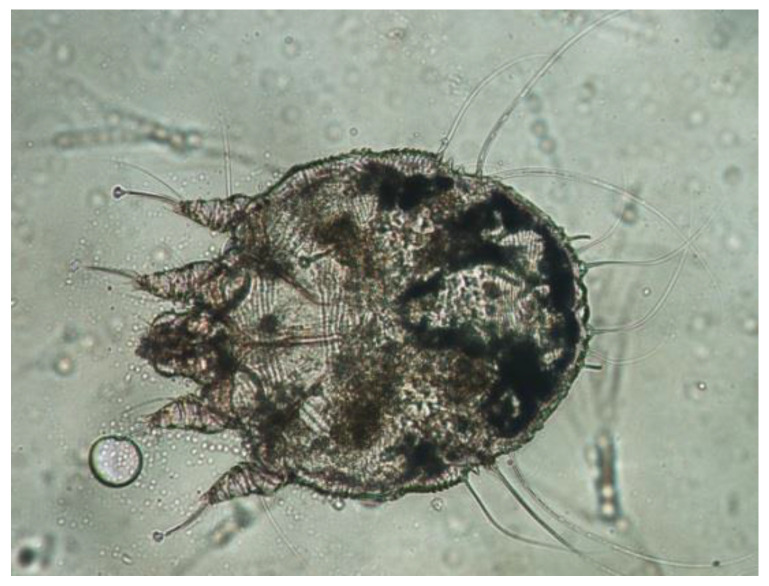
*Sarcoptes scabiei* visualized under a light microscope.

**Figure 3:**
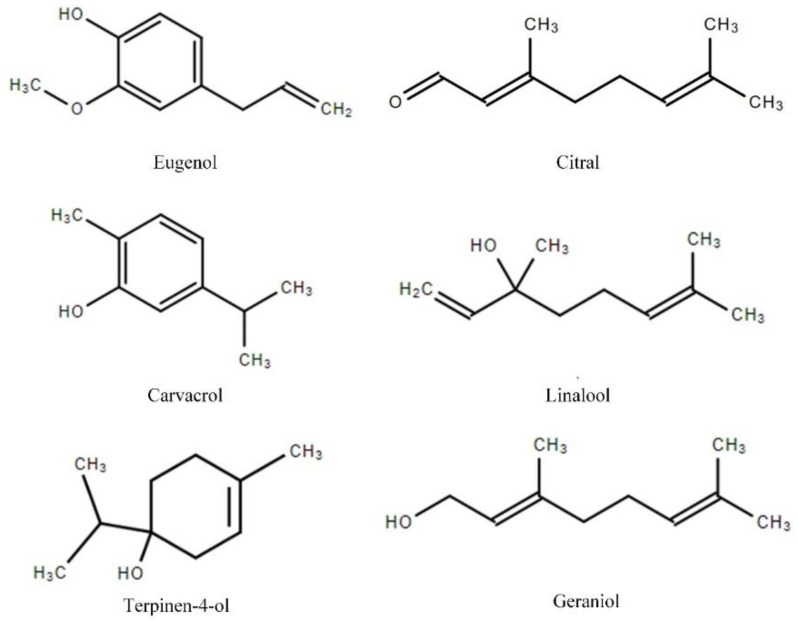
Molecule structures of the six terpenes.

The *Sarcoptes* mites used in the experiments were collected from the crusts of naturally infested New Zealand white rabbits in a rabbit farm in Nanning, Guangxi Province, China. Before the beginning of the study, we contacted the farm owners and obtained their permission to have the infected rabbits involved. The study protocol was approved by the ethics committee of Guangxi University (approval no. GXU2019-019). The crusts were placed in petri dishes and transported to the laboratory within a few hours. The *Sarcoptes* mites (Figure 2) were isolated one by one with a needle for further testing under a stereomicroscope. Six terpenes, namely terpinen-4-ol, citral, linalool, eugenol, geraniol, and carvacrol (Figure 3), were purchased from Shanghai Macklin, Shanghai, China. All of the compounds were of the highest purity available (from 95 to 99%). Benzyl benzoate was obtained from the biochemical company Shanghai Aladdin, Shanghai, China, and used as a positive control.

### 3.2. In Vitro Evaluation of Miticidal Activity

The six terpenes were tested against *S. scabiei* at concentrations of 5%, 1%, and 0.5% by diluting them in paraffin oil. Ten female mites were exposed to the treatments in 3 cm petri dishes and were treated with a total volume of 0.5 mL of the products, as previously described [16]. Based on the good efficacies of carvacrol, eugenol, and geraniol in the above tests, the three terpenes were further assessed to calculate the LC_50_ (median lethal concentration) in 30 min. Five concentration gradients were set for each terpene. Paraffin oil was used as a negative control. The positive control was treated with 25% benzyl benzoate. All petri dishes were incubated at 25 °C and 70% relative humidity. All of the tests were replicated five times. Immobility of the mites, a lack of reactions, or persistent immobility within 1 min following stimulation with a needle were considered fatal.

### 3.3. Statistical Analysis

The data were analyzed using SPSS, version 20.0. The median lethal time (LT_50_) and the lethal concentration (LC_50_, LC_90_) values were calculated by probit regression. The survival curves were calculated using the Kaplan–Meier method. The differences in the survival curves were assessed using the logrank (Mantel–Cox) test and expressed as *p* values. Values of *p* < 0.05 were considered significant.

## 4. Discussion

Several essential oils have been proven as promising alternatives for the control of *Sarcoptes* mites, but the commercial development of such products requires an assessment of the efficacy of the essential oils’ components, individually or in combination. The six terpenes tested in the present study are the major components of the essential oils that display significant activity against *S. scabiei*. Among the terpenes tested here, carvacrol stands out as the most active against *Sarcoptes* mites. In the present study, it killed all mites within 17 min at a concentration of 0.5%.

Carvacrol is a monoterpenic phenol with a variety of biological properties, including antioxidant, antibacterial, antifungal, anticancer, anti-inflammatory, hepatoprotective, spasmolytic, and vasorelaxant [23]. A previous study demonstrated that carvacrol at a concentration of 0.63% was able to kill *Psoroptes ovis* after 12 h [22]. This inconsistency in miticidal efficacy could be attributed to the differences in the tested mite species [24].

Eugenol was found to be the main component in clove, tulsi, and cinnamon oils, which showed remarkable miticidal activity [16,18]. Eugenol has been demonstrated to possess antibacterial, antiviral, antifungal, anticancer, anti-inflammatory, and antioxidant properties [25]. Our study demonstrated that the LC_50_ value of eugenol against *Sarcoptes* mites at 30 min was 0.79%, while the LC_50_ value at 1 h was about 0.2% (13.0 mM), as reported by Pasay et al. [17]. The lesser efficacy of eugenol may be due to the mites developing some drug resistance from the rabbits that were regularly treated with ivermectin. It was reported that the drug-resistant mites are less susceptible to essential oils than the naïve ones [17]. Among the terpenes tested here, carvacrol and eugenol had strong activity against *S. scabiei*. The two compounds share the same phenolic hydroxyl group in their structures (Figure 3). This specific structure may play an important role in the miticidal process via acting on the GABA and octopamine receptors [26]. Other terpenes, such as thymol, which share the same phenolic hydroxyl group, might exhibit potential miticidal activity and are worth testing.

Geraniol, which is a widely used fragrance terpene found in citronella oil, rose oil, and palmarosa oil, has a number of pharmacological properties, including antitumor, anti-inflammatory, antioxidative, and antimicrobial activities, as well as hepatoprotective, cardioprotective, and neuroprotective effects [27]. Previous studies have reported that geraniol was active against mites (*Dermanyssus gallinae* and *Psoroptes cuniculi*), ticks, and head lice [20,28,29,30]. In the present study, at a concentration of 1%, geraniol killed all mites in 24 min, while the same concentration killed all *P. cuniculi* within 3.25 min [20].

Terpinen-4-ol, citral, and linalool at a concentration of 5% showed better efficacy than 25% benzyl benzoate, but at lower concentrations, the three terpenes displayed limited effects on the mites. Walton et al. [14] demonstrated that the LT_50_ value for 2.1% terpinen-4-ol against *Sarcoptes* mites was 35 min. Citral, the major component of lemongrass oil, was lethal to two-spotted spider mites (*Tetranychus urticae*) [31]. Perrucci et al. [32] showed that 3% linalool applied twice a week for 3 weeks resulted in 80% of rabbits being free of *Psoroptes* mites.

The effects of essential oils and their components on insects have been investigated in several studies. Many essential oils have been shown to exhibit neurotoxicity by targeting arthropods’ nervous systems, including the enzyme acetylcholinesterase (AChE) [33,34], ionotropic GABA receptors [35], and octopamine receptors [36]. Some terpenes, such as carvacrol, thymol, and eugenol, have been found to have neuroinhibitory effects, while linalool produces excitatory effects [37]. The lipophilic nature of essential oils is thought to play an important role in penetrating through the cuticle of arthropods [37]. However, the effectiveness of terpenes as insecticides may be limited by the insect’s metabolic detoxification system [38].

## 5. Conclusions

In summary, carvacrol, eugenol, and geraniol showed prominent acaricidal efficacies. Considering their significant activities against *S. scabiei* eggs [39], the three terpenes represent promising leads for the development of topical acaricides. Further investigations are needed to fully understand the mechanisms underlying the effects of terpenes and evaluate whether they have synergistic effects on mites.

## Figures and Tables

**Table 1:** Probit regression analysis of the toxicity (LT_50_) of six terpenes against *Sarcoptes* mites in vitro.

Compound	LT_50_ (95%FL *)		
	5%	1%	0.5%
Terpinen-4-ol	18.63 min (18.86–20.91)	6.07 h (5.31–6.82)	22.25 h (19.40–26.54)
Citral	11.97 min (10.49–13.21)	3.87 h (3.37–4.33)	6.11 h (5.32–6.83)
Linalool	26.29 min (23.44–28.73)	8.84 h (6.84–11.12)	39.94 h (28.17–111.09)
Eugenol	2.06 min ^#^	6.41 min (4.29–7.87)	56.28 min (50.74–62.53)
Geraniol	3.73 min ^#^	24.83 min (17.54–30.68)	1.75 h (1.02–2.22)
Carvacrol	1.03 min ^#^	3.45 min ^#^	6.74 min ^#^

* 95% confidence limits. ^#^ no 95% confidence limits.

**Table 2:** Concentrations of three terpenes required to kill 50% and 90% of *Sarcoptes* mites at 30 min postexposure.

Compound	LC50 (%)	95%CI	LC90 (%)	95%CI
Carvacrol	0.24	0.23–0.25	0.33	0.20–0.37
Eugenol	0.79	0.76–0.81	0.99	0.93–1.11
Geraniol	0.91	0.87–0.95	1.26	1.16–1.46

## Data Availability

Not applicable.
